# Epigenetic Priming of Bladder Cancer Cells With Decitabine Increases Cytotoxicity of Human EGFR and CD44v6 CAR Engineered T-Cells

**DOI:** 10.3389/fimmu.2021.782448

**Published:** 2021-11-17

**Authors:** Camilla M. Grunewald, Corinna Haist, Carolin König, Patrick Petzsch, Arthur Bister, Elfriede Nößner, Constanze Wiek, Kathrin Scheckenbach, Karl Köhrer, Günter Niegisch, Helmut Hanenberg, Michèle J. Hoffmann

**Affiliations:** ^1^ Department of Urology, Medical Faculty, Heinrich Heine University Duesseldorf, Duesseldorf, Germany; ^2^ Department of Otorhinolaryngology and Head and Neck Surgery, Medical Faculty, Heinrich Heine University Duesseldorf, Duesseldorf, Germany; ^3^ Department of Pediatrics III, University Children’s Hospital Essen, University of Duisburg-Essen, Essen, Germany; ^4^ Biological and Medical Research Center (BMFZ), Heinrich Heine University Duesseldorf, Duesseldorf, Germany; ^5^ Immunoanalytics: Tissue Control of Immunocytes, German Research Center for Environmental Health, Helmholtz Zentrum München, Munich, Germany

**Keywords:** epigenetic inhibitors, bladder cancer, chimeric antigen receptor, immunotherapy, T-cell

## Abstract

**Background:**

Treatment of B-cell malignancies with CD19-directed chimeric antigen receptor (CAR) T-cells marked a new era in immunotherapy, which yet has to be successfully adopted to solid cancers. Epigenetic inhibitors of DNA methyltransferases (DNMTi) and histone deacetylases (HDACi) can induce broad changes in gene expression of malignant cells, thus making these inhibitors interesting combination partners for immunotherapeutic approaches.

**Methods:**

Urothelial carcinoma cell lines (UCC) and benign uroepithelial HBLAK cells pretreated with the DNMTi decitabine or the HDACi romidepsin were co-incubated with CAR T-cells directed against EGFR or CD44v6, and subsequent cytotoxicity assays were performed. Effects on T-cell cytotoxicity and surface antigen expression on UCC were determined by flow cytometry. We also performed next-generation mRNA sequencing of inhibitor-treated UCC and siRNA-mediated knockdown of potential regulators of CAR T-cell killing.

**Results:**

Exposure to decitabine but not romidepsin enhanced CAR T-cell cytotoxicity towards all UCC lines, but not towards the benign HBLAK cells. Increased killing could neither be attributed to enhanced target antigen expression (EGFR and CD44v6) nor fully explained by changes in the T-cell ligands PD-L1, PD-L2, ICAM-1, or CD95. Instead, gene expression analysis suggested that regulators of cell survival and apoptosis were differentially induced by the treatment. Decitabine altered the balance between survival and apoptosis factors towards an apoptosis-sensitive state associated with increased CAR T-cell killing, while romidepsin, at least partially, tilted this balance in the opposite direction. Knockdown experiments with siRNA in UCC confirmed BID and BCL2L1/BCLX as two key factors for the altered susceptibility of the UCC.

**Conclusion:**

Our data suggest that the combination of decitabine with CAR T-cell therapy is an attractive novel therapeutic approach to enhance tumor-specific killing of bladder cancer. Since BID and BCL2L1 are essential determinants for the susceptibility of a wide variety of malignant cells, their targeting might be additionally suitable for combination with immunotherapies, e.g., CAR T-cells or checkpoint inhibitors in other malignancies.

## Introduction

In recent years, the field of tumor immunotherapy has evolved rapidly (1). One of the most exciting approaches is the use of autologous patient-derived T-cells that have been genetically modified to express chimeric antigen receptors (CARs). Such CAR molecules combine the antigen-binding properties of monoclonal antibodies with the lytic capacity of T-cells (2). Remarkable remission rates in clinical trials using CAR T-cells directed against CD19^+^ B-cell malignancies led to FDA approval of the first CAR T-cell therapies for patients with relapsed/refractory acute lymphoblastic leukemia (ALL) or diffuse large B-cell lymphoma (DLBCL) and for patients with primary mediastinal B-cell lymphoma (PMBCL) in 2017. In addition to Yescarta (axicabtagene ciloleucel) and Kymriah (tisagenlecleucel), two other CD19-targeted CAR T-cell therapies, namely, Tecartus (brexuscabtagene autoleucel) and Breyanzi (lisocatbagene maraleucel), were approved in 2020 and 2021, respectively. Finally, the first BCMA-targeted CAR T-cell therapy, Abecma (idecabtagene vicleucel), for relapsed/refractory multiple myeloma received approval in March 2021 (3).

The exceptional success in hematological malignancies could not be transferred to solid cancers, due to issues with T-cell trafficking, immunosuppressive tumor microenvironment, target antigen heterogeneity, and intrinsic regulatory mechanisms of T-cells in these malignancies (4). Although no definite clinical data on CAR T-cell therapy has been published yet for its use in bladder cancer, several early phase I/II clinical trials are ongoing targeting the prostate-specific membrane antigen (PSMA), the human epidermal growth factor receptor 2 (HER2), Nectin4/FAP, NKG2D ligands, and the receptor tyrosine kinase-like orphan receptor 2 (ROR2), respectively (NCT03185468, NCT03740256, NCT 03932565, NCT03018405, NCT03960060^1^).

Epigenetic dysregulation caused by DNA hypermethylation through DNA methyltransferases (DNMTs) and histone hypoacetylation catalyzed by histone deacetylases (HDACs) leads to silencing of key genes and thereby determines the phenotype of urothelial carcinoma of the bladder (UC) with regard to pathogenesis, tumor biology, and outcome to standard treatment (5). Novel therapeutic strategies directed towards these epigenetic drivers include inhibitors of DNMTs (DNMTi, e.g., decitabine) and HDACs (HDACi, e.g., romidepsin). Importantly, both of these drugs were already approved for the treatment of certain hematological malignancies. Besides induction of broad gene expression changes affecting various cellular processes, epigenetic inhibitors (epidrugs) can also remodel the differentiation and the immune phenotypes of both cancer and immune cells (6, 7). Epidrugs can also influence key components of apoptosis signaling in cells, e.g., expression of the FAS receptor (CD95) is regulated by DNA methylation (8). Concurringly, epidrugs have been shown to resensitize tumors to previously failed therapies and to affect both the cancer cells as well as the tumor microenvironment (9). We therefore considered it an interesting approach to prime UC for immune-oncological approaches like CAR T-cell therapy, especially as we previously characterized the functional importance of individual HDAC isoenzymes and their potential as therapeutic targets in UC (10). So far, only a few clinical trials evaluate the use of epidrugs in bladder cancer as part of combination therapies (11).

To determine whether epigenetic pretreatment of UC cell lines (UCC) might affect their susceptibility towards cytotoxic killing by CAR T-cells, we developed a combined treatment protocol involving UCC treatment with either the DNMTi decitabine (DEC) or the HDACi romidepsin (ROM). We used previously established treatment conditions of 3 nM ROM for 3 days (10), whereas DEC was applied in a low-dose/long-term protocol (100 nM DEC for 7 days), following emerging data that administration of low doses of an DNMTi might enhance the desired epigenetic effects whilst reducing toxicity (12) being comparably well tolerated in patients, e.g., with low-risk myelodysplastic syndrome (13). As target antigens, we chose two different surface molecules on UCC: The epidermal growth factor receptor (EGFR) is a transmembrane glycoprotein and member of the ERBB family of surface proteins that are associated with cell migration, adhesion, and proliferation. EGFR expression is highly enriched in UC tissues and strongly associated with certain tumor grades and stages as well as risk of recurrence (14). Although there is no data for CAR T-cells directed against EGFR in UC, cetuximab as the most prominent anti-EGFR antibody is currently discussed as radiosensitizer (15), albeit prior phase II data on the use of cetuximab in metastatic UC showed limited activity of the antibody as a single modality treatment (16). As a second target antigen structure, we selected CD44v6, a splicing variant of the cell surface adhesion receptor CD44, that is overexpressed on a large variety of malignant cells (17). CD44v6 expression is associated with tumor cell invasion, metastasis, and disease progression and has been correlated with increased tumor grade and stage in UC (18). Similar to EGFR, clinically relevant expression of CD44v6 on normal epithelial tissue has mainly been reported in skin and oral mucosa (19).

In this study, we identified CD44v6 and EGFR as promising target antigens for CAR T-cell therapy of UC and demonstrated that the specific killing capacity of CAR T-cells against malignant UCC is strongly influenced by expression of pro- and anti-apoptotic genes in the malignant cells that can be modulated by epigenetic treatment strategies.

## Materials and Methods

### Cell Culture

We used four UCC, namely, RT-112, BFTC905, VM-CUB-1, and UM-UC-3 (10). HBLAK was used as normal urothelial control cell line (20). All cell lines were regularly authenticated by STR profiling, checked for mycoplasma contamination, and cultured as described (21). Human embryonic kidney cells (HEK293T) were obtained from DSMZ (Braunschweig, Germany) and cultured in DMEM GlutaMAX supplemented with 10% fetal bovine serum (FBS) and 1% penicillin/streptomycin (P/S) (all Thermo Fisher Scientific, Schwerte, Germany).

Human peripheral blood mononuclear cells (PBMCs) were isolated from peripheral blood of healthy adult volunteers by density-gradient centrifugation (Cytiva, Marlborough, MA, USA). Blood donors gave informed consent according to the protocol (#2019-623) approved by the local ethics committee/IRB in Düsseldorf. Prior to transduction, T-cells were prestimulated with immobilized antibodies against CD3 (OKT3, Ortho Biotech, Neuss, Germany) and CD28 (BD Biosciences Pharmingen, San Diego, CA, USA), as well as 100 IU/ml Interleukin-2 (IL-2, Proleukin, Novartis, Basel, Switzerland) in IMDM (Sigma, MO, USA) containing 10% FBS, 1% P/S, 1% glutamine as previously described (22, 23).

### *In Vitro* Treatment of Cell Lines With DEC or ROM and siRNA Transfection

5-Aza-2’-Desoxycytidin (DEC) was purchased from Sigma-Aldrich (Steinheim, Germany) and romidepsin (ROM) from Selleckchem (Houston, TX, USA) and dissolved in DMSO. Control cells were treated with corresponding DMSO concentrations. Since DEC is known to have only an *in vitro* half-life of 5–16 h at 37°C, 100 nM DEC was freshly added every 24 h for 3 days during medium change. Hereafter, cells were cultured for 4 additional days, washed, and passaged into 96-well plates prior to co-culture with CAR T-cells (24). For ROM treatment, cells were cultured in the same medium containing 3 nM ROM for 3 days according to common ROM treatment protocols (25). ROM solution is stable at room temperature for about 24 h. Thus, we expected the T-cells not to be touched by active ROM when these were added to treated UCC for co-culture after 72 h. As a control, we performed washout experiments demonstrating no difference between samples with or without ROM washout 72 h after UCC treatment before adding T-cells for co-culture.

UCC were transfected with siRNA as described (21) using Lipofectamine RNAiMAX (Thermo Fisher Scientific) and 8 nM of the ON-TARGETplus SMARTPool (Dharmacon, GE Healthcare, Freiburg, Germany), comprising a set of four individual siRNAs against each of the targets TRADD, DAXX, BCL2L1, and BID or the non-targeting control pool (**Supplementary Table S1**).

### Flow Cytometry

Surface expression of antigens was assessed 3 days (ROM) and 7 days (DEC) after treatment by immunofluorescence staining and flow cytometry (MACSQuant Analyzer 10; Miltenyi Biotec, Bergisch Gladbach, Germany). Likewise, T-cell phenotype was determined by immunofluorescence staining with subsequent flow cytometric analysis. Antibodies and counterstaining are listed in **Supplementary Table S1**. Data were analyzed using the FlowJo™ Software (v10.0.7). Results were expressed as percentage of positive cells and median fluorescence intensity (MFI). Values of unstained cells were subtracted from values of stained cells.

### Generation of Lentiviral Constructs and T-Cell Transduction, Selection, and Functional Assays

The CD19 and CD44v6 CAR constructs were described previously (26–29). The EGFR CAR lentiviral vector contains optimized sequences for the heavy-chain and light-chain variable region segments derived from the monoclonal antibody of Cetuximab (30). In comparison to a Cetuximab-based CAR construct published previously (31), the light chain of Cetuximab in our construct was shortened at amino acid (aa) 108 and the heavy chain at aa 128 (Haist et al. submitted). HEK293T cells were transfected with polyethylenimine (PEI, Sigma-Aldrich, St. Louis, MO, USA) using 6 µg HIV1 helper plasmid (gag-pol-rev), 6 µg vesicular stomatitis virus glycoprotein (VSV-G) envelope, and 6 µg lentiviral CAR construct plasmid for the generation of recombinant lentiviral particles (26). The next day, the medium was changed to IMDM, and then after additional 16–20 h and 0.45 µm filtration, the culture supernatants were directly used for transduction.

After prestimulation on immobilized CD3/CD28 monoclonal antibodies, T-cells were transduced with lentiviral particles on the recombinant fibronectin fragment Retronectin^®^ as described (26, 32). Subsequently, T-cells were cultured in medium containing 100 IU/ml IL-2 for 72 h. To obtain >98% pure CAR T-cells, transduced T-cells were incubated with magnetic microbeads coupled to the CD34 QBEND10 antibody (Miltenyi Biotec) (**Supplementary Table S1**), which recognizes a 99 amino acid sequence that we have included as novel hinge domain in our CARs (29) (Bister et al., in press; patent EP3293199). Subsequently, CD34 microbead-stained T-cells were purified on MACS MS columns according to the manufacturer’s instructions (Miltenyi Biotec).

For cytotoxicity assays, UCC were pretreated with DEC or ROM or cultured in the presence of DMSO and seeded in U-bottom 96-well plates. On day 3 of ROM and day 7 of DEC pretreatment or day 2 after siRNA transfection, cells were counted and CD19, EGFR, and CD44v6 CAR T-cells added at different effector to target (E:T) cell ratios (3:1, 1:1, 0.3:1, 0.1:1, 0.03:1, and 0.01:1). Prestimulated non-transduced T-cells served as additional controls. After 16 h of co-culture at 37°C, the non-adherent T-cells and the dead tumor cells were carefully removed by washing steps. Remaining adherent UCCs were incubated with the CellTiter 96^®^ AQueous One Solution Cell Proliferation substrate according to manufacturer specifications (Promega, Fitchburg, WI, USA), and viability was determined on a TECAN sunrise (Tecan Group AG, Männedorf, Switzerland). The percent lysis was calculated as follows:


100%−(Absorption of targets incubated with T-cells/Absorption of reference targets)×100

For the spheroid model, 1 × 10^5^ untreated or DEC-treated UM-UC-3 or BFTC905 cells were seeded in ultra-low attachment U-bottom 96-well plates (Corning, Wiesbaden, Germany). Twenty-four hours later, spheroid formation was confirmed by microscope, and CD19, EGFR, CD44v6 CAR T-cells or prestimulated non-transduced T-cells from three different donors were added at E:T ratios of 1:1, 1:2, or 1:4. After 16 h of co-culture, CellTiter-Glo^®^ 3D Cell Viability substrate (Promega) was added according to manufacturer specifications and viability determined on a Wallac VICTOR 2 (Perkin-Elmer, Waltham, MA, USA). The percent specific lysis was calculated as follows:


100%−(Luminescence of spheroids with CAR T-cells/Luminescence of spheroids with non-transduced T-cells)×100

To analyze cytokine secretion by T-cells, supernatants of the wells containing the 1:1 E:T ratio of effector to target cells were harvested and stored at −20°C. Then 50 µl of each supernatant was analyzed using the MACSPlex Cytotoxic T/NK cell kit (Miltenyi Biotec) and measured on a MACSQuant Analyzer 10 according to the manufacturer’s instructions.

### Next-Generation RNA Sequencing

Total RNA was extracted from BFTC905 and UM-UC-3 cells treated with 100 nM DEC using the RNAeasy Mini Kit (Qiagen, Hilden, Germany). DMSO-treated cells were harvested in parallel as controls. RNA was quantified using Qubit RNA HS Assays (Thermo Fisher Scientific). Quality was confirmed by capillary electrophoresis using Fragment Analyzer and Total RNA Standard Sensitivity Assay (Agilent Technologies, Santa Clara, CA, USA). Library preparation and next-generation sequencing were performed as described (21). Multigroup comparisons were calculated using the Empirical Analysis of DGE (version 1.1, cutoff = 5) after grouping of samples (three biological replicates each) according to their respective experimental condition. The resulting p-values were adjusted for multiple testing by FDR and Bonferroni-correction. A p-value of ≤ 0.05 was considered significant. Cutoff for differential gene expression was set to 1.5-fold. Further analysis and data visualization were performed using Microsoft Excel and Graph Pad Prism 8. Venn diagrams were prepared with the online tool Venny 2.0 (33). GO group analysis was performed using the online tool DAVID (34).

### Western Blot Analysis

Proteins were extracted 72 h after siRNA transfection and used for Western Blot analysis as described (21). Expression of knockdown targets was detected by antibodies listed in **Supplementary Table S1**.

### Statistical Analysis

All experiments were repeated at least three times. Significance between groups was analyzed by means of Graph Pad Prism 9 using 2-way ANOVA (analysis of variances) with Dunnett’s correction for multiple comparison. P-values of < 0.05 were considered significant and denoted with an asterisk.

## Results

### The CAR Target Antigens EGFR and CD44v6 Are Expressed on Urothelial Cells

**Figure 1:**
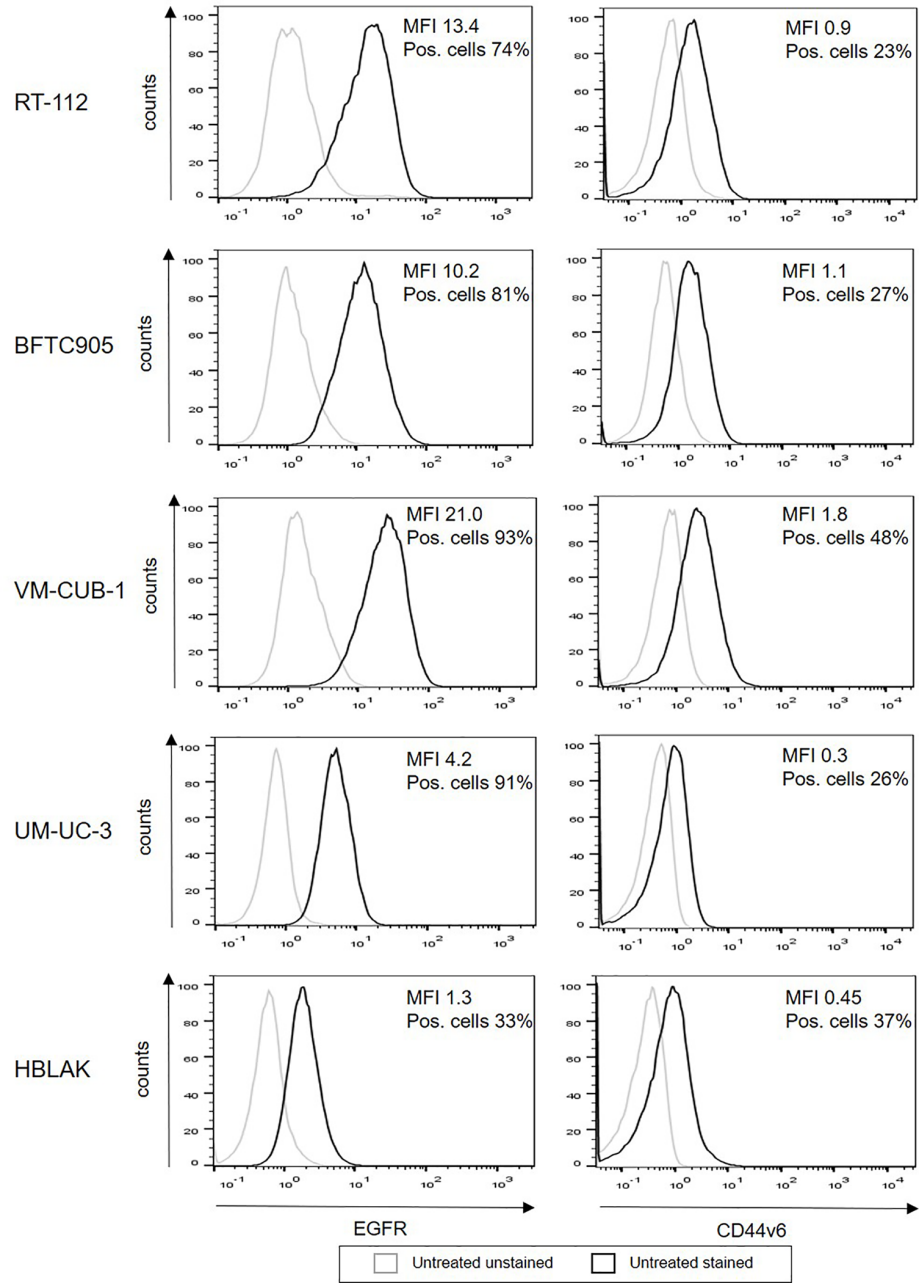
Surface expression of EGFR and CD44v6 on untreated UCC and HBLAK. The UCC RT-112, BFTC905, VM-CUB-1, UM-UC-3, and benign HBLAK were stained with the indicated antibodies and analyzed by flow cytometry. The figure shows representative histograms out of at least three independent experiments for each cell line. Histogram of unstained cells appear in gray, stained cells in black.

In order to determine the role of epigenetic treatment for immunotherapy of bladder cancer, we used a representative set of human urothelial carcinoma cell lines, RT-112, BFTC905, VM-CUB-1, and UM-UC-3, which covers the heterogeneity of urothelial cancer. HBLAK (20), a non-malignant urothelial cell line, was employed as control. First, we evaluated surface expression of two potentially suitable target antigens on our cell lines, EGFR and CD44v6. All UCC expressed high levels of EGFR (MFI 4.2-21.0, 74–93% positive cells), while the expression level was clearly lower on HBLAK cells (MFI 1.3, 33% positive cells, **Figure 1**). In contrast, CD44v6 expression was similar for all five cell lines (MFI 0.3–1.8, 23–48% positive cells).

### High-Level Expression of EGFR and CD44v6 CARs on Normal Allogeneic T-Cells After Lentiviral Transduction Followed by MACS Enrichment

**Figure 2:**
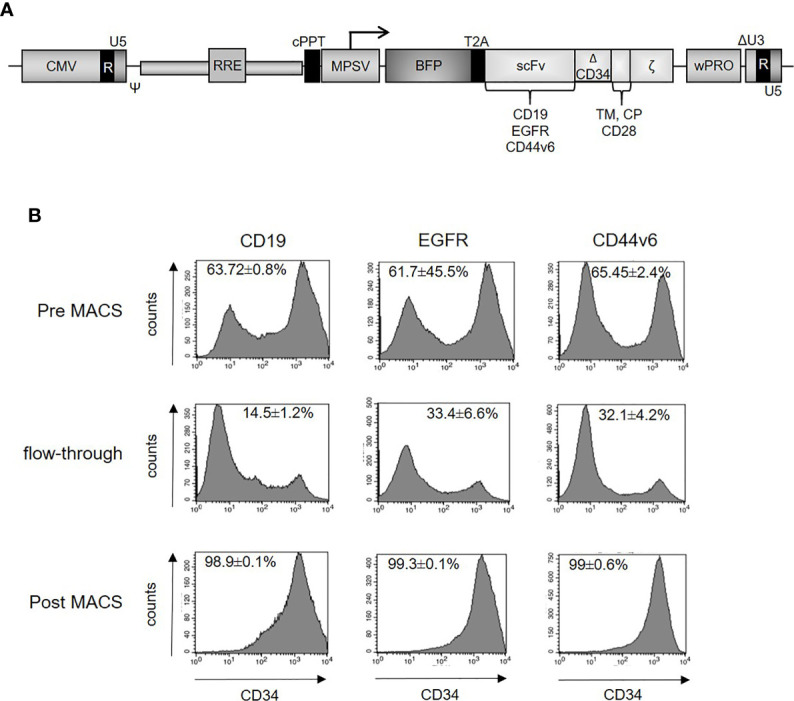
**(A)** Schematic design of the lentiviral vector for expression of the second-generation CARs. The constructs contain CD19, EGFR or CD44v6 scFv, CD34 hinge domain, CD28 transmembrane and intracellular domain, CD3ζ cytosolic domain and blue fluorescent protein (BFP). **(B)** Enrichment of CAR-positive T-cells. Primary human T-cells were lentivirally transduced with constructs expressing CD19, EGFR, or CD44v6 CARs. Three days after transduction, CAR-positive T-cells were stained with CD34 microbeads (Miltenyi) and finally enriched by magnetic cell sorting. Different fractions (pre, flow-through, post MACS) were analyzed by flow cytometry using the QBEND-PE antibody (**Supplementary Table S1**). CAR expression (x-axis) is plotted against the absolute cell count (y-axis). Illustrations were gated with the percentage of positive cells. Data are depicted as mean ± SEM for three different experiments.

To generate CAR T-cells, we employed previously established CARs against EGFR, CD44v6, and CD19 (29) (Haist et al. submitted). The standard lentiviral vectors (26) expressed second-generation CARs containing the CD8 leader peptide, the single-chain variable fragments (scFvs) of a EGFR-, CD44v6-, or CD19-specific monoclonal antibody, an extracellular hinge region using 99 amino acids from human CD34 (29), the CD28 transmembrane and co-stimulatory domains as well as the CD3ζ signaling domain **(** **Figure 2A** **)**. Three days after transduction, CAR-expressing T-cells were purified. Representative samples for the purification steps (Pre MACS, flow-through, Post MACS) were analyzed by flow cytometry (**Figure 2B**), demonstrating transduction efficiencies between 61.7 and 65.5% prior to enrichment and ≥98% strongly CAR positive T-cells after the enrichment step. MACS-selected CAR T-cells were further characterized for the CD4/CD8 ratio and the memory phenotype by flow cytometry. While the phenotype of CD19, EGFR, and CD44v6 CAR T-cells did not differ from the phenotype of non-transduced T-cells expanded under identical conditions in parallel (**Supplementary Figures S1A, B**), it is noteworthy that the majority of the T-cells were central memory cells (CM; **Supplementary Figure S1B**), as defined by co-expression of CD62L and CD45RO (35).

### Primary Human T-Cells Expressing EGFR and CD44v6 CARs Effectively Kill UCC

**Figure 3:**
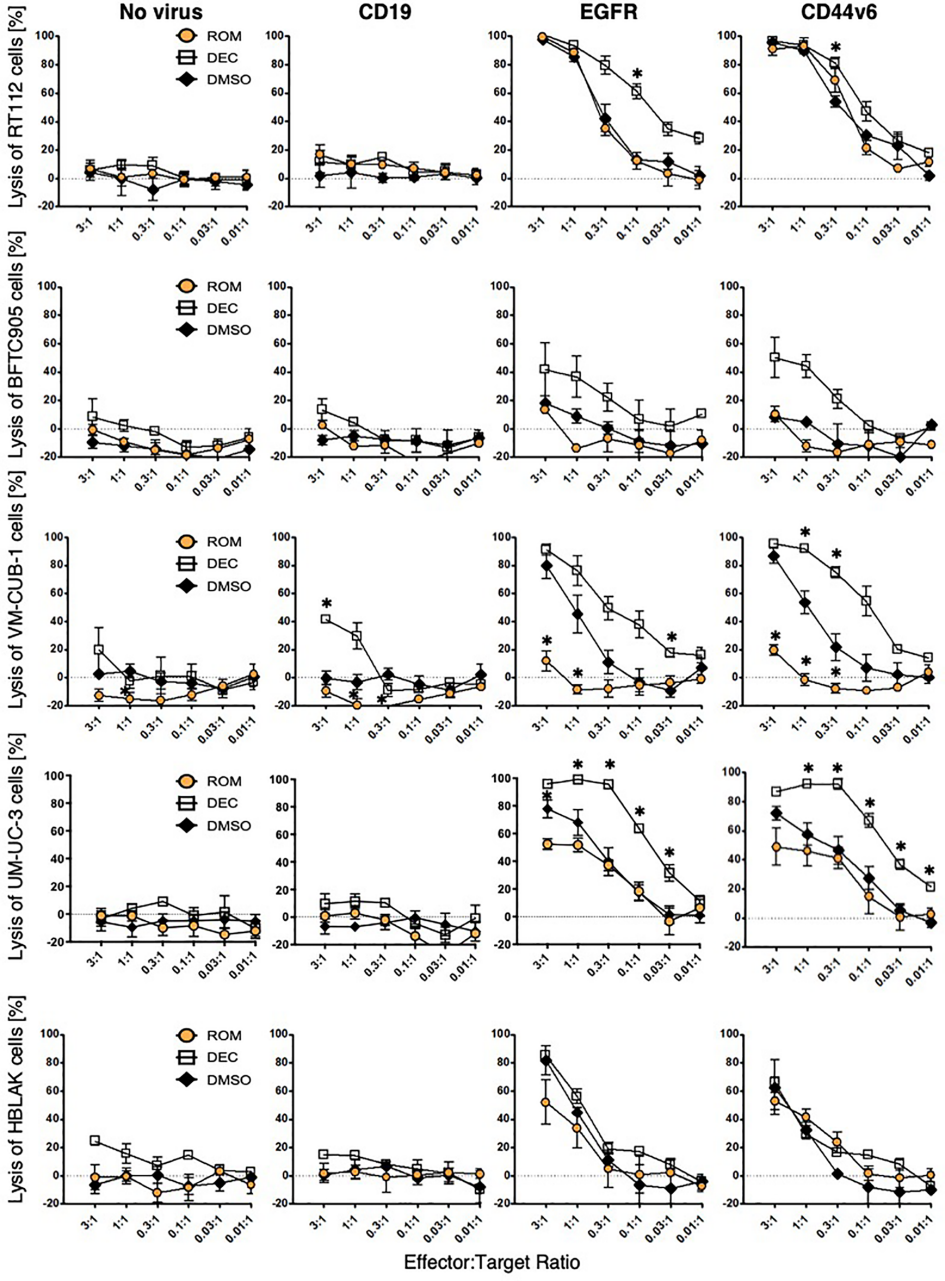
CAR T-cell cytotoxicity can be modulated by epigenetic treatment. UCC and HBLAK cells were pretreated with 100 nM decitabine (white square), 3 nM romidepsin (orange circle), or DMSO (black rhombus) and subsequently co-cultured with CD19-, EGFR-, or CD44v6 CAR T-cells or untransduced (no virus) T-cells. After 16 h co-culture, lysis was determined by MTT assay. Graphs depict the mean values ( ± SD) for percentage of lysed cells from at least three independent experiments. Statistical significance (p-value <0.05) is denoted by asterisk and was determined by two-way ANOVA and Dunnett’s correction for multiple comparisons.

To assess the specific cytotoxicity, ≥98% MACS-enriched EGFR and CD44v6 CAR T-cells were co-incubated with the four UCC and HBLAK in different E:T ratios (3:1 to 0.01:1). Negative controls in these assays were non-transduced T-cells as well as CD19 CAR T-cells. We initially confirmed that our flow cytometry-based (26) and our 96-well plate cytotoxicity assay (29) provided similar results (**Supplementary Figure S2A**). As the plate cytotoxicity assay facilitated a much higher throughput of samples, all consecutive cytotoxicity assays were analyzed with this methodology. The lysis curves obtained with the 96-well plate assay revealed that the EGFR and CD44v6 CAR T-cells efficiently and specifically killed RT-112, VM-CUB-1, and UM-UC-3 cells to similar degrees at comparable E:T ratios (**Figure 3**). Notably, BFTC905 cells, despite robustly expressing both target antigens (MFI 10.2 and 1.1, 81% and 27% positive cells, respectively) were killed less efficiently. HBLAK cells were killed more efficiently at higher effector-to-target-cell ratios (1:1 and 3:1), despite having low target antigen expression levels (MFI 1.3 and 0.45, 33% and 37% positive cells, respectively). Therefore, these results clearly demonstrated that the degree of CAR T-cell cytotoxicity is not simply dependent on the expression level of the target antigens.

### Epigenetic Treatment of UCC Influences CAR T-Cell Cytotoxicity

To investigate whether cytotoxicity of CAR T-cells can be influenced by treatment of the target cells with epidrugs, UCC and HBLAK were pretreated with either DEC, ROM, or DMSO as control and subsequently incubated with CAR T-cells. Viability analysis revealed that DEC pretreatment (white square) sensitized all four UCC more towards EGFR and CD44v6 CAR T-cell-mediated cytotoxicity when compared to DMSO-treated cells (black rhombus) (**Figure 3**). Especially BFTC905 cells, which were insufficiently killed by EGFR and CD44v6 CAR T-cells, were considerably better killed after DEC with killing values augmented from 18 ± 5 to 42 ± 19% (EGFR) and from 8 ± 2 to 47 ± 14% (CD44v6) at the 3:1 ratio, respectively. UM-UC-3 and VM-CUB-1 cells were also killed considerably more effective when pretreated with DEC. Cytotoxicity of HBLAK cells was not clearly affected by pretreatment with neither inhibitor, suggesting increased tumor-specific killing of CAR T-cells after DEC treatment. In contrast, ROM treatment (orange circle) resulted in unchanged (BFTC905, RT-112) or even decreased (UM-UC-3, VM-CUB-1) specific cytotoxic activity for both CAR T-cells. Even though ROM is not stable for 72 h incubation time, we performed additional washout experiments to demonstrate that reduced cytotoxicity after ROM pretreatment did not result from negative impact of the compound on CAR T-cells (**Supplementary Figure S2B**).

Cancer cell spheroids can partially fill the gap between conventional 2D *in vitro* assays and animal models, as these spheroids better model the infiltration of immune effector cells into solid cancer tissues (36). To confirm our findings in a more advanced model, we therefore used DEC-treated and untreated UM-UC-3 and BFTC905 spheroids and performed subsequent cytotoxicity assays after co-incubation with EGFR, CD44v6, and CD19 CAR or non-transduced T-cells, similar to the 2D experiments described above. As shown in **Supplementary Figures S3A, B**, EGFR and CD44v6 CAR T-cells killed cells in the spheroids of both cell lines at comparable efficiencies, as observed in the 2D *in vitro* cultures (**Figure 3**). However, the effects for DEC-pretreated BFTC905 were generally less pronounced. The secretion profiles for granzyme B, TNFα, and GM-CSF in the culture supernatants very well reflected the excellent killing of UM-UC-3 cells and the much lower killing of BFTC905 cells by EGFR and CD44v6 CAR T-cells, while the cytokine profiles for CD19 CAR T-cells were similar to those of non-transduced T-cells (**Supplementary Figures S3C, D** **).** Importantly, the DEC pretreatment of both UC cell lines did not result in increased cytokine secretions by the CAR T-cells, suggesting that the increased lysis is more likely due to a tumor-intrinsic than a T-cell-mediated effect.

### Expression Levels of the Target Antigens, Immune Checkpoints, and Adhesion Molecules Do Not Correlate Well With the Killing Efficacy

**Figure 4:**
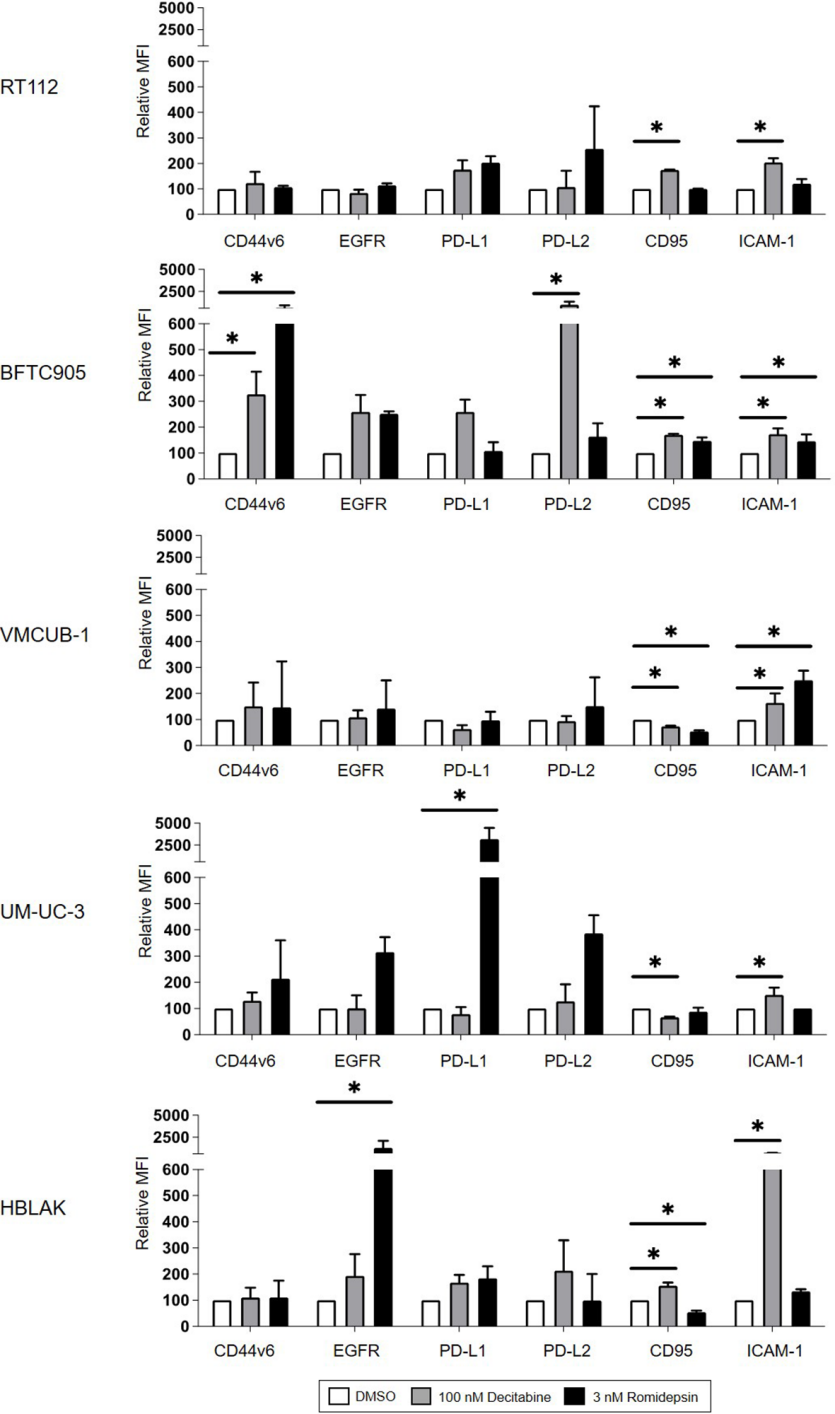
Cell surface protein expression of CD44v6, EGFR, PD-L1, PD-L2, CD95, and ICAM-1 on target cells after DMSO, DEC, or ROM treatment. The graphs represent changes in median fluorescence intensity (MFI) of each marker normalized to DMSO control. White bars represent the values for DMSO-treated cells, and gray and black bars represent results of DEC- or ROM-treated cells, respectively. Results are shown as the mean ( ± SD) of at least three independent experiments; values of unstained cells are subtracted. p-values were calculated by means of two-way ANOVA and subsequent Dunnett’s correction for multiple comparisons. Statistical significance (p-value ≤ 0.05) is denoted by asterisk.

Increased target antigen expression would provide a straightforward explanation for enhanced CAR T-cell cytotoxicity following DEC treatment. EGFR and CD44v6 expression levels increased strongly in BFTC905 cells after DEC treatment (**Figure 4**), which correlated well with the improved killing observed with both CAR T-cells. However, despite an even stronger increase in CD44v6 expression and comparable EGFR expression after ROM compared to DEC treatment, no enhanced killing was detected when using ROM-treated BFTC905 cells. RT112 cells were killed to similar degrees after all treatments by both CARs, in correlation to the unaltered target expression levels. Simultaneously, we observed increases in EGFR surface expression after ROM treatment in UM-UC-3 cells, but reduced cytotoxicity. Therefore, the increased cytotoxicity after DEC pretreatment cannot simply be explained by increased levels of target antigen expression.

It is well established that inhibitory immune checkpoints can regulate CAR T-cell cytotoxicity (37). We therefore analyzed whether treatment with DEC or ROM led to altered expression of PD-L1 or PD-L2. As shown in **Figure 4**, UM-UC-3 cells showed increased expression of PD-L1 and PD-L2 after ROM treatment compared to DEC or DMSO. This increase inversely correlated with the decrease in CAR T-cell cytotoxicity after ROM treatment. In contrast, DEC pretreatment of BFTC905 cells increased the PD-L1 and PD-L2, and ROM, the PD-L2 expression levels. Therefore, the higher expression of inhibitory checkpoint molecules did not explain the increased cytotoxicity observed after DEC but not after ROM pretreatment.

Recent work from Kantari-Mimoun et al. on CAR T-cell function indicated that the adhesion molecule ICAM-1 (intracellular adhesion receptor-1) might be involved in the recognition of malignant cells by CAR T-cells (38). ICAM-1 expression was significantly induced in all cell lines by DEC and in VM-CUB1 and BFTC905 cells also by ROM (**Figure 4**). While this increase may have contributed to enhanced CAR T-cell lysis, it did not appear to be an important determinant for the cytotoxicity, since DEC and ROM pretreatment both caused increased ICAM-1 expression in BFTC905 cells, whereas killing was only improved by DEC.

Finally, the FAS (CD95) and FASL axis is another mechanistic pathway by which CAR T-cells can mediate tumor cell killing (39). Accordingly, we detected a significant induction of CD95 expression after DEC treatment in RT-112, BFTC905, and HBLAK cells (**Figure 4**), which only partially explained the variations in the cytotoxicity curves (**Figure 3**).

### Epigenetic Treatment Alters the Balance of Survival and Apoptosis Signaling in UCC

To identify other target cell factors that might contribute to the differences in CAR T-cell cytotoxicity, we performed next-generation RNA sequencing of BFTC905 and UM-UC-3 cells after treatment with DEC. High-throughput data from UM-UC-3 and VMCUB-1 cells treated with 3 nM ROM for 72 h were already available from an earlier publication (10) (GEO accession GSE70120).

Overall, 1,553 genes were differentially expressed in BFCT905 cells after 7 days of DEC treatment compared to only 927 genes in UM-UC-3 cells. A comparable number of genes was downregulated in both cell lines after DEC treatment (**Supplementary Figures S4A, B**). To identify immune response-associated factors that were altered by epidrugs, we developed a list of candidate factors (n=143) based on literature reviews (40). However, only few of these candidate genes significantly deviated in their expression upon DEC treatment (1.5-fold change, p ≤ 0.05), and none of the genes with robust changes could easily explain the altered CAR T-cell killing efficacy (**Supplementary Figure S4C**).

**Figure 5:**
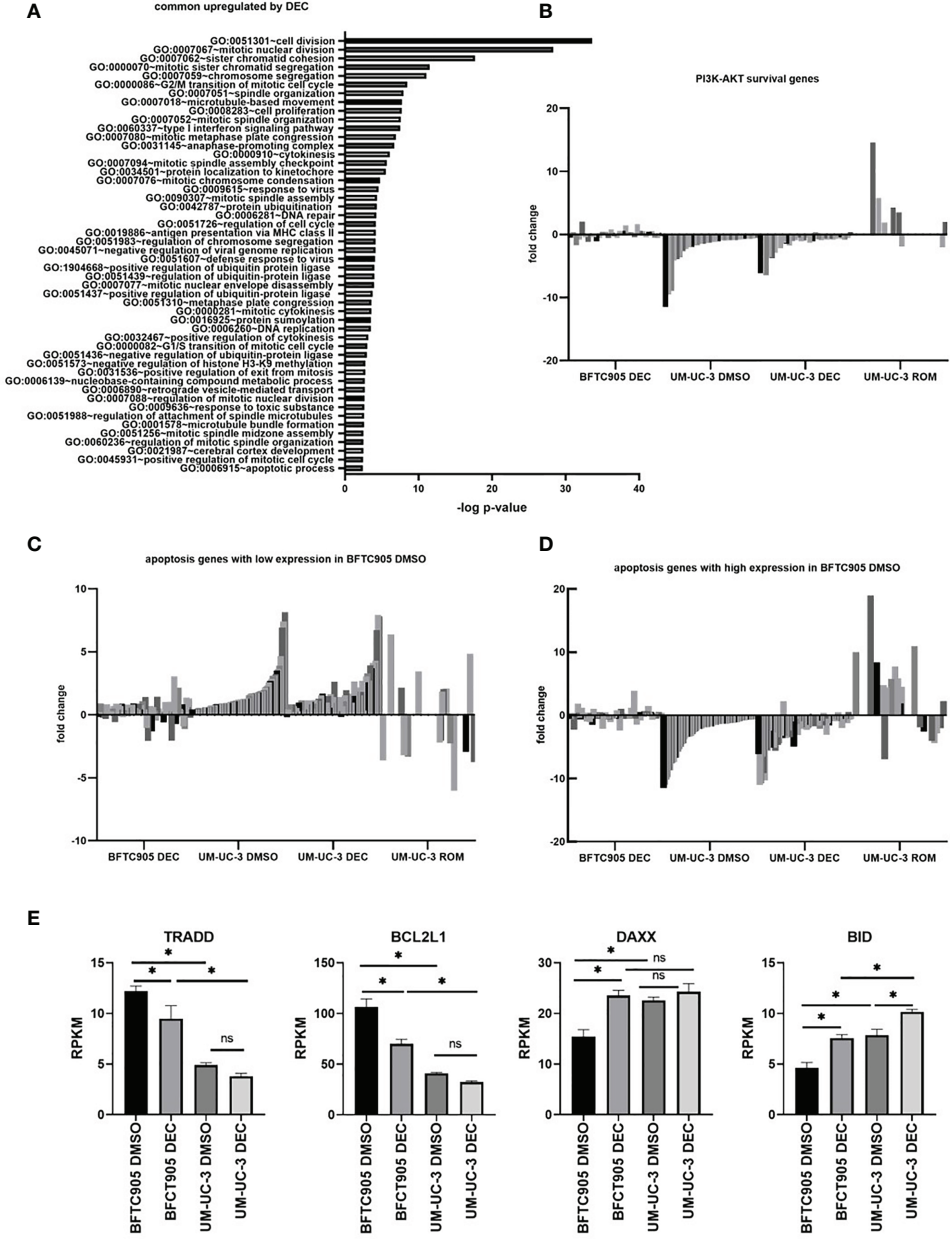
Gene expression changes in UCC BFTC905 and UM-UC-3 after DEC treatment determined by RNA sequencing. **(A)** GO group analysis was performed for 207 genes commonly induced by DEC in both UCC (d7) using the online tool DAVID (34, 41). Only the GOs with p<0.004 are displayed. **(B)** Fold change expression of PI3K-AKT survival genes (shades of gray) in indicated samples relative to BFTC905 DMSO used for normalization. Gene lists were downloaded from the Broad Institute GSEA gene sets database (https://www.gsea-msigdb.org/gsea/msigdb/genesets.jsp?collection=CP : KEGG). Genes names and expression values are given in **Supplementary Table S6**. **(C)** Fold change expression of apoptosis genes in indicated samples relative to BFTC905 DMSO. Apoptosis genes with lower expression in BFTC905 DMSO compared to UM-UC-3 DMSO are displayed. See **Supplementary Table S7** for gene names and expression values. **(D)** Fold change expression of apoptosis genes in indicated samples relative to BFTC905 DMSO. Apoptosis genes with higher expression in BFTC905 DMSO compared to UM-UC-3 DMSO are displayed. See **Supplementary Table S8** for gene names and expression values. **(E)** Mean RPKM expression values from RNA sequencing analysis obtained from three replicates are visualized as bar graphs for the four apoptosis regulators that were chosen for further functional analysis by siRNA knockdown. Statistical significance (p-value <0.05) is denoted by asterisk and was determined by two-way ANOVA and Dunnett’s correction for multiple comparisons. ns, not significant.

To analyze the effect of DEC treatment in an unbiased way, we determined the overlap of differentially expressed genes between both cell lines and performed GO analysis on this gene set **(** **Supplementary Figure S4B**, **Figure 5A** and **Supplementary Tables S2–5** **)**. This overlap of genes was surprisingly small, and enrichment in GOs could not explain differences in CAR T-cell killing (**Supplementary Tables S2–5**). However, we found intrinsic transcriptomic differences between BFTC905 and UM-UC-3 cells that could affect the balance between cell survival and apoptosis. Untreated BFTC905 cells had a more pronounced pro-survival and anti-apoptotic profile compared to UM-UC-3 cells. These findings could readily explain why untreated UM-UC-3 cells were better killed by CAR T-cells.

Using GSEA gene set analysis, we merged the candidate gene lists for PI3K-AKT survival and apoptosis signaling (extrinsic and intrinsic), where genes could be either induced or downregulated, depending on their pro- or anti-apoptotic functions, and displayed fold change expression after DEC and ROM treatment as heatmaps. As shown in **Supplementary Figure S5**, many genes that were induced or remained unchanged by DEC were downregulated by ROM and vice versa. Especially survival and apoptosis genes were differentially altered by DEC and ROM, and differences in gene expression between BFTC905 and UM-UC-3 cells became more obvious after DEC treatment. We next divided the differentially expressed apoptosis-related genes into two groups: Genes in the first group were expressed at lower levels in DMSO-treated BFTC905 compared to DMSO-exposed UM-UC-3 cells. Their induction by DEC might therefore be responsible for pro-apoptotic effects associated with the increased killing of UCC by CAR T-cells after DEC treatment. The second group included apoptosis genes that were at least twofold higher expressed in DMSO-treated BFTC905 cells compared to UM-UC-3 and therefore might exert anti-apoptotic functions, thus at least partially providing protection against CAR T-cell-mediated killing. In order to visualize the differences between the two cell lines and treatments in a bar diagram, the expression levels were normalized to the values of BFTC905 DMSO-treated cells. We also displayed the differentially expressed survival genes in a similar manner. Our analyses clearly demonstrated a difference between BFTC905 and UM-UC-3 cells with regard to expression of cell survival genes (**Figure 5B**, see **Supplementary Table S6** for gene names and detailed expression levels). Survival genes were more strongly expressed in BFTC905 cells compared to UM-UC-3 cells and only marginally induced by DEC in both cell lines. In contrast, pro-apoptotic genes, which were weakly expressed in untreated BFTC905 cells compared to UM-UC-3, were indeed upregulated by DEC treatment in both cell lines (**Figure 5C** and **Supplementary Table S7**), thus providing an explanation why this treatment was associated with improved CAR T-cell killing of UCC. In contrast, ROM treatment of UM-UC-3 cells strongly induced pro-survival signaling genes (**Figure 5B**) and reduced some genes from the pro-apoptotic group (**Figure 5C**), which would explain the reduced CAR T-cell killing of ROM pre-treated UCC. The second group of apoptosis genes (**Figure 5D** and **Supplementary Table S8** **)** was more strongly expressed in untreated BFTC905 cells compared to UM-UC-3 cells, presumably protecting them from apoptosis. Many of them were downregulated by DEC in both UCC, thereby potentially facilitating cell death induction. These genes responded conversely to ROM, thus more likely protecting the cells from the CAR T-cell cytotoxicity.

Based on these *in silico* analyses, the different killing efficacies can be explained by differential changes in the balance between survival and apoptosis induced by the two epigenetic inhibitors. To mechanistically confirm the functional role of apoptosis regulators in determining the susceptibility of DEC-treated UCC toward CAR T-cell-induced cell death, we selected four candidates from the RNA sequencing data for further knockdown experiments (**Supplementary Tables S7, 8**). These candidates were chosen considering their regulatory function in intrinsic and extrinsic apoptosis signaling, their expression differences between untreated BFTC905 and UM-UC-3 cells, and also their response to DEC treatment. We chose two anti-apoptotic genes with higher expression in BFTC905 cells compared to UM-UC-3 that were reduced in expression by DEC, *TRADD* and *BCL2L1* with the latter encoding for BCLX (**Figure 5E** **)**. Likewise, we chose two pro-apoptotic genes that were weakly expressed in BFTC905 compared to UM-UC-3 cells and that were induced by DEC, *DAXX* and *BID* (**Figure 5E** **)**.

### BCL2L1 and BID Are Important Target Cell Susceptibility Factors for CAR T-Cell Killing

Next, we performed siRNA knockdown of these four apoptosis regulators. We transfected UM-UC-3 cells with all four siRNAs pools as well as a pool of non-targeting control siRNAs. BFTC905 cells were only transfected with TRADD, BCL2L1, and the control siRNA pool, as the already low CAR T-cell cytotoxicity for unmodified BFTC905 (**Figure 3**) would not have permitted to reliably detect further diminished cytotoxic activity. The two siRNA-transfected UCC were submitted to CAR T-cell cytotoxicity assays and harvested on the same day for Western Blot analysis.

**Figure 6:**
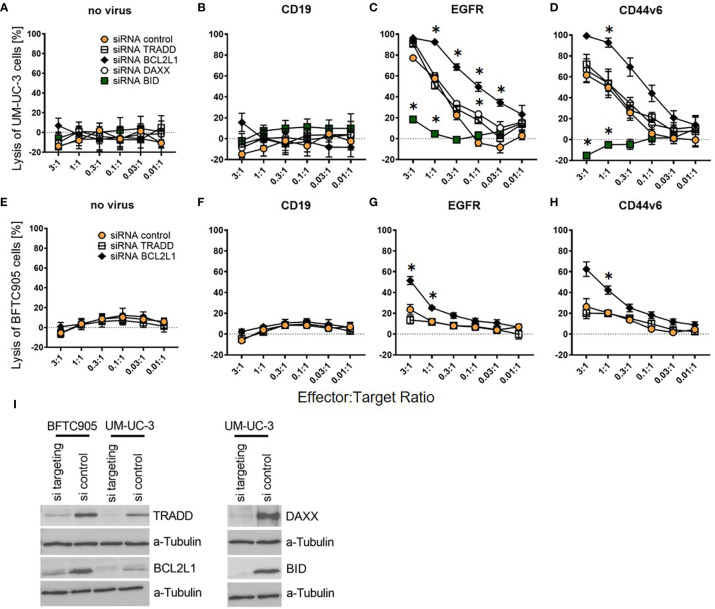
Killing efficiency of EGFR and CD44v6 CAR T-cells against UM-UC-3 and BFTC905 cells after siRNA knockdown of apoptosis regulating genes. **(A)** Cytotoxicity of untransduced (no virus), CD19, EGFR, and CD44v6 CAR T-cells against UM-UC-3 **(A–D)** and BFTC905 **(E–H)** cells transfected with indicated siRNA was determined. Graphs depict the mean values ( ± SD) of three independent experiments. Values for different effector to target ratios (E:T ratio 3:1–0.01:1) are given. **(I)** Western Blot analysis of TRADD (34 kDa), BCL2L1 (26 kDa), BID (22 kDa), and DAXX (110 kDa) protein expression after the respective siRNA knockdown (si targeting) compared to control cells (si control). Statistical significance (p-value <0.05) is denoted by asterisk and was determined by two-way ANOVA and Dunnett’s correction for multiple comparisons.

Cytotoxicity analysis demonstrated that knockdown of anti-apoptotic BCL2L1 mRNA (black rhombus) strongly increased the killing of UM-UC-3 and BFTC905 cells, while the TRADD knockdown (white square) had no effect (**Figures 6C, D, G, H** **)**. Notably, while the pro-apoptotic DAXX knockdown (white circle) did not alter cytotoxicity of CAR T-cells towards UM-UC-3 cells (**Figures 6C, D** **)**, the BID knockdown (green square) almost completely abolished the cytotoxicity of CAR T-cells for UM-UC-3 cells (**Figures 6C, D** **)**. Negative controls were not significantly affected by knockdown (**Figures 6A, B, E, F** **)**. Hence, two of the four candidate genes, *BCL2L1* and *BID*, were identified as essential determinants for the susceptibility of DEC-pretreated UCC towards CAR T-cell-induced cytotoxicity, thus providing a compelling new approach to influence the susceptibility of UCC for immunotherapy strategies. Western blot analysis revealed highly efficient knockdown for TRADD, BCL2L1, DAXX, and BID (**Figure 6I**).

## Discussion

Epigenetic changes are characteristic for all cancers and essential for accretion of the 10 properties of malignant cells proposed as hallmarks of cancer, which also includes anti-apoptotic signaling (42). Therefore, our main objective here was to investigate whether pretreatment of cancer cells with epidrugs improves the cytotoxicity and target specificity of immunotherapy with CAR T-cells as effector cells.

As the search for ideal target antigens to achieve specific antitumor activity still is a major challenge for solid cancers, we evaluated well-established surface molecules, EGFR and CD44v6, as possible target antigens for CAR T-cell therapy of UC. Both antigens are overexpressed on solid cancers (17, 19), and we already had constructed second-generation CARs in lentiviral vectors that efficiently killed human head and neck squamous cell carcinomas (29) (Haist et al. submitted). Using a set of UCC we demonstrated that EGFR was robustly expressed on all malignant UCC and that the expression levels were higher compared to non-malignant uroepithelial HBLAK cells. CD44v6 was also expressed on all cell lines, albeit with comparable levels between UCC and HBLAK. EGFR and CD44v6 CAR T-cells efficiently killed the cells of three UCC and also HBLAK, although the killing of cells of the non-malignant cell line was less efficient and required higher E:T ratios. We also noted that the cancer line BFTC905, even though it robustly expressed both target antigens, was only marginally killed by the EGFR and CD44v6 CAR T-cells from several donors. In conclusion, the killing efficacy of CAR T-cells did not correlate well with the target antigen expression patterns on the UC cell lines and therefore had to be influenced/determined by other mechanisms.

Multiple lines of evidence demonstrate that epidrugs like DNMTi (DEC) and HDACi (ROM) can exhibit profound immune-modulating effects on several levels (40, 43, 44), including both epigenetic modulation of the immunosuppressive tumor microenvironment as well as direct modulation of tumor cells. However, in order to clearly identify the cellular pathway in the tumor cells responsible for the susceptibility to CAR T-cells, we pretreated the UCC for several days with the epidrugs and then only later added the transduced immune effector cells for the overnight cytotoxic assays. Interestingly, DEC pretreatment increased the CAR T-cell cytotoxicity towards all UCC, but not towards the non-malignant HBLAK cells, thus suggesting a tumor-specific modulation of gene expression in malignant cells. This malignant cell-specific increased killing is an important finding here, as *on-target off-tumor* effects due to expression of the target antigen on normal cells is a well-recognized problem/side-effect of CAR T-cell therapy and poses a significant clinical challenge. Major strategies currently pursued to increase the tumor specificity of CAR T-cell therapy are the use of CARs with reduced affinity, which might not kill normal cells with lower target antigen expression levels, or injection of high-affinity CAR T-cells directly into the tumor tissue (31, 45, 46). The specific effect of demethylating agents on malignant cells that we describe here might be another relatively simple way to increase the *on-tumor* effects of CAR T-cells. We have confirmed the DEC sensitizing effects with two different CARs, EGFR and CD44v6, for multiple UCC in 2D and for two also in 3D cultures, achieving comparable killing. Noteworthy is also that for some of the DEC-pretreated cell lines, the control T-cells (no virus, CD19) demonstrated slightly increased killing efficacy at higher effector-to-target ratios. This non-specific cytotoxicity most likely occurred due to the maximum stimulation of T-cells prior to transduction and was not caused by a direct effect of DEC, as due to passaging of pretreated UCC and also the short half-life of DEC in culture (24), the T-cells were never in contact with the compound.

We next wanted to decipher the mechanism responsible for the improved killing efficacy after DEC treatment in all UCC and to understand why untreated BFTC905 cells were hardly killed, despite their strong target antigen expression comparable to the other UCC. Therefore, we analyzed the expression of molecules that are known to influence the interaction between T-cells and cancer cells. Importantly, the expression of the two target antigens did not consistently increase in the examined cell lines and therefore could not explain the improved killing after DEC. Most strikingly, both target antigens increased under the epigenetic treatments in BFTC905 cells; however, killing was only improved by exposure to DEC and not to ROM. Although expression of PD-L1, PD-L2, and ICAM-1 increased after treatment with DEC, we could not detect a consistent correlation between CAR T-cell cytotoxicity and changes in the protein expression levels as assessed by flow cytometry. T-cell cytotoxicity is partially mediated by the interaction between FAS ligand on T-cells and the death receptor FAS (CD95) on the malignant cells (39). FAS was similarly expressed on all UCC at baseline and further increased by DEC in individual cell lines. In particular, BFTC905 cells responded to both epigenetic inhibitors by increasing the protein expression, but were more sensitive to CAR T-cells after DEC and less after ROM treatment.

For a more unbiased screening approach to identify factors determining the susceptibility towards CAR T-cell cytotoxicity, we performed RNA sequencing analysis of two DEC-treated UCC, UM-UC-3 as a representative candidate for the effects of DEC and ROM and BFTC905 as outlier with almost no CAR T-cell killing at baseline. The GO term analysis demonstrated that DEC shifted the balance between survival/pro- and anti-apoptotic genes towards an expression profile that could facilitate the induction of cell death by cytotoxic CAR T-cells. ROM treatment shifted this balance into the opposite direction, thus providing a possible explanation for its poor effects on CAR T-cell killing efficacy. Although Yang et al. also reported divergent effects of DEC and ROM on apoptosis-related genes in metastatic human colon carcinoma cells (47), the impact of these substances on other cancer entities appeared to be cancer-type specific: ROM treatment induced G2/M phase arrest and apoptosis *via* activation of ERK-MAPK and JNK-MAPK pathways in hepatocellular cancer (48), while ROM induced apoptosis in non-small cell lung cancer cells by inhibition of RAF-MEK-ERK PI3K/AKT signaling and by downregulation of anti-apoptotic genes and upregulation of the pro-apoptotic BAX (49).

Based on our *in silico* analysis, we chose four genes that were strongly deregulated by DEC treatment and performed specific siRNA knockdown in BFTC905 and UM-UC-3 cells. The co-culture of treated cells with EGFR and CD44v6 CAR T-cells identified BCL2L1 and BID, both members of the BCL-2 family of death regulators, as key cellular factors whose modulation can ameliorate the T-cell cytotoxicity towards UCC. Mechanistically, pretreatment of UCC with DEC induced the expression of the pro-apoptotic BID, which can counterbalance the function of anti-apoptotic BCL-2 like proteins (50), thus promoting increased cytotoxicity of CAR T-cells. Concurringly, knockdown of BID in the target cells by siRNA rendered them resistant to CAR T-cells. In addition, DEC reduced the expression of anti-apoptotic BCL2L1 (also known as BCLX), thereby further altering the balance towards apoptosis induction by CAR T-cells. Concurringly, siRNA knockdown of BCL2L1 improved the CAR T-cell cytotoxicity similar to DEC pretreatment of UCC, particularly of BFTC905 cells.

Due to our study design, the CAR T-cells were not exposed to DEC. This is reflected by the findings that the cytokine profiles in the supernatants of the spheroid experiments were similar for the DEC-treated and untreated cells, when using the CAR T-cells with *on-target* CARs. Although BFTC905 cells were not efficiently killed, the induction of granzyme B and TNFa over the control wells clearly indicated that the EGFR and CD44v6 CAR T-cells specifically recognized their target antigens. Obviously, pretreatment of tumor cells with DEC prior to CAR T-cell therapy does not reflect the *in vivo* situation in patients, as epidrugs will also directly affect the CAR T-cells themselves and indirectly will alternate the whole tumor microenvironment (TME) (7). However, based on the mechanistic study performed here, demonstrating that DEC treatment sensitizes UC tumor cells towards CAR T-cell cytotoxicity, the combination of both approaches appears attractive. Importantly, the effects of low-dose DEC treatment on CAR T-cells were already reported to be promising, namely, augmenting cytokine production and increased lytic antitumor activity of CAR T-cells *in vitro* and *in vivo*. In addition, DEC induces the expression of memory-related genes and reduction of exhaustion-related genes. Inhibition of epigenetic modifiers is also associated with long-persisting CAR T-cells due to enrichment of a memory-like phenotype (7, 51). Finally, low-dose DEC treatment may also positively influence the TME *via*, e.g., the induction of chemokines like CXCL9 and -10, thereby promoting T-cell infiltration (52), or *via* the decrease of immunosuppressive cells in the microenvironment like myeloid-derived suppressor cells (MDSCs) (53). Thus, systemic low-dose DEC treatment, such as already used for MDS treatment (13), will most likely have benefits *in vivo* beyond the direct influence on the UC.

In conclusion, our study provides an exciting rationale for combining DEC with CAR T-cell therapy for bladder cancer. To our knowledge, only one study using murine solid cancer cells reported that siRNA interference with BCL-2, BCLXL, or BAX can determine the sensitivity of solid tumor cells towards cytotoxicity of T-cells (54). Interestingly, loss of BID, together with FADD (Fas associated *via* death domain) and TRAIL (tumor necrosis factor-related apoptosis-inducing ligand), was reported to contribute to resistance of lymphoma cells to CD19 CAR T-cells (55). Nevertheless, considering the role of BCL-2 family members in B-cell lymphoma therapy (56), it is not surprising that combinations of CAR T-cell therapy with compounds targeting apoptosis regulators, like BH3 mimetics (e.g., ABT-737) or pan-BCL-2 inhibitors, are currently discussed for mature B-cell malignancies. Our results provide a rationale for extending this combinatorial approach to solid cancers in the future, especially if members of the BCL-2 family are dysregulated in the malignant cells (50, 57).

## Data Availability Statement

Next generation sequence data is available *via* the GEO repository (accession number GSE164862). All other data relevant to this study is included in the article or uploaded as **Supplementary Material**.

## Author Contributions

CG, CH, MH, HH, GN, and EN contributed to conception and design of the study. CG, CH, MH, CK, AB, PP, and KK performed experiments and statistical analyses. CG wrote the first draft of the manuscript. MH, CH, HH, GN, CW, and KS wrote sections of the manuscript. All authors contributed to manuscript revision, read, and approved the submitted version.

## Funding

This work was supported, in part, by the Foschungskommission of the Medical Faculty of the Heinrich Heine University Duesseldorf to CG, the Duesseldorf School of Oncology DSO to AB, and the European Regional Development Fund (EFRE-0801320, iCAN33) to HH.

## Conflict of Interest

The authors declare that the research was conducted in the absence of any commercial or financial relationships that could be construed as a potential conflict of interest.

## Publisher’s Note

All claims expressed in this article are solely those of the authors and do not necessarily represent those of their affiliated organizations, or those of the publisher, the editors and the reviewers. Any product that may be evaluated in this article, or claim that may be made by its manufacturer, is not guaranteed or endorsed by the publisher.

## Footnotes

1
www.clinicaltrials.gov
