# Supplementary material for: Epigenetic Priming of Bladder Cancer Cells With Decitabine Increases Cytotoxicity of Human EGFR and CD44v6 CAR Engineered T-Cells
Source: Front Immunol. 2021 Nov 17;12:782448. doi: 10.3389/fimmu.2021.782448 (PMC8637820; doi:10.3389/fimmu.2021.782448)
Supplement: Supplementary file 1 [file DataSheet_1.docx]

Supplementary Figures


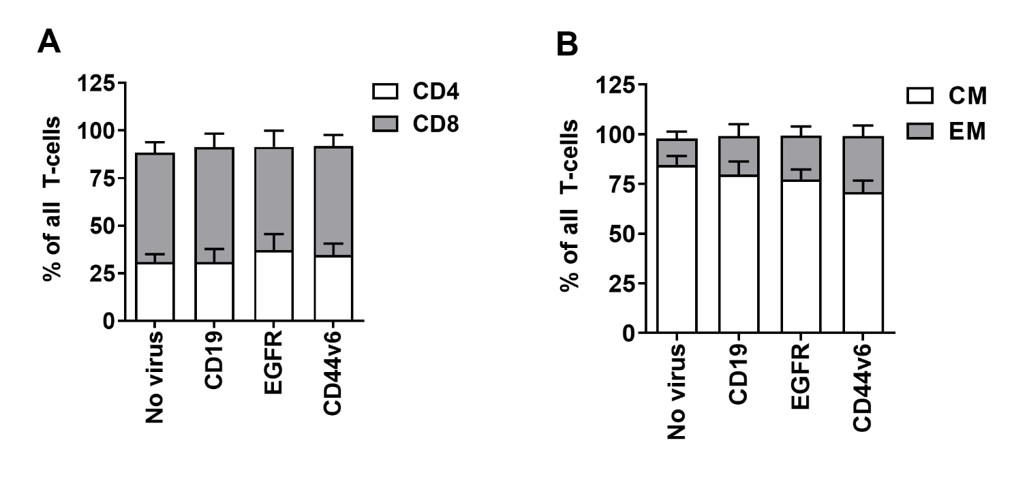


**Supplementary Figure S1.** Phenotype characterization of CAR and untransduced T-cells. MACS-enriched CAR and untransduced T-cells were flow cytometrically analyzed for (A) helper (CD4), cytotoxic (CD8), (B) central memory (CM, CD62L+ CD45RO+) and effector memory (EM, CD62L- CD45RO+) phenotype. Percentage values are represented as mean ± SEM.


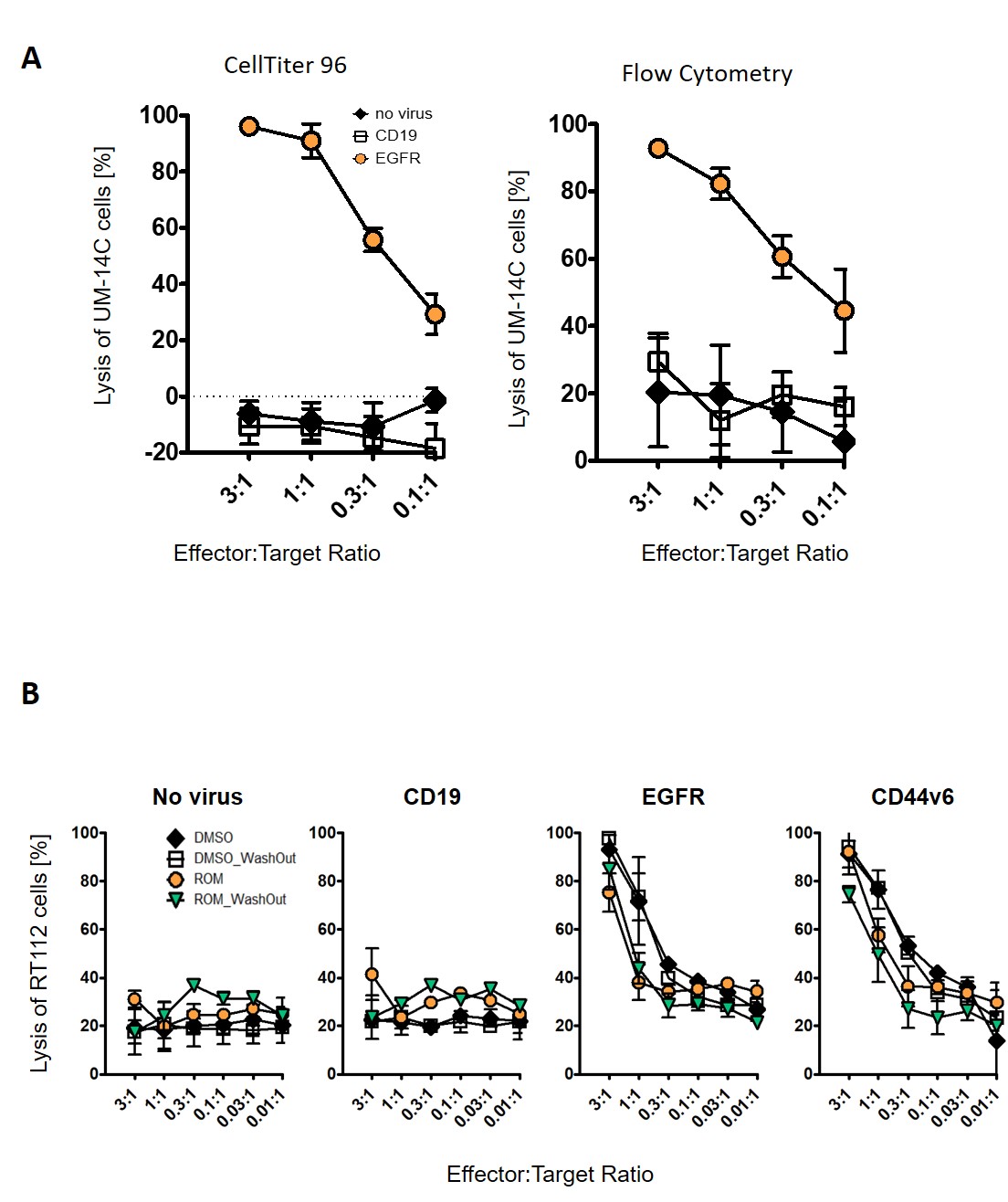
**Supplementary Figure S2.** Validation experiments for experimental setup. (A) To determine the assay of choice for measuring CAR T-cell induced cytotoxicity results of viability assay (left panel) were compared with flow cytometry measurements (right panel). Graphs depict the mean values (±SD) for percentage of lysed cells. Black rhombus represents untransduced control, white square symbols represent the values CD19 controls, orange circle values for EGFR CAR. (B) To check for any effects of residual ROM on depicted CAR T- cells, ROM pretreated RT112 were either washed (green triangle) or not washed (orange circle) prior to co-culture with CAR T-cells. DMSO treated controls were handled likewise (white square and black rhombus). Graphs depict the mean values (±SD) for percentage of lysed cells.

**
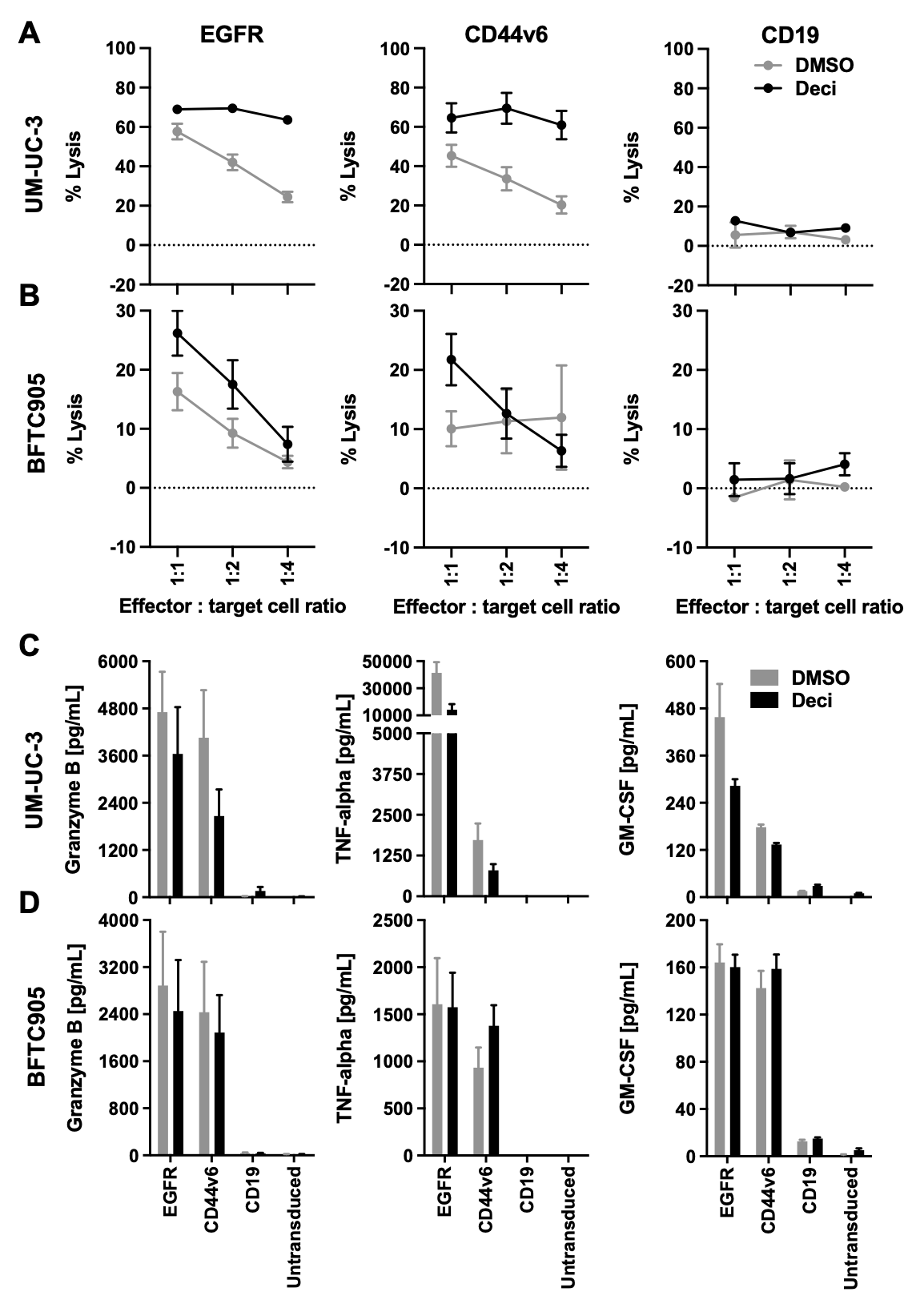
Supplementary Figure S3.**  CAR T-cell cytotoxicity and cytokine secretion against UM-UC-3 and BFTC905 spheroids. 100 nM DEC (black) and DSMO (grey) pretreated UM-UC-3 and BFTC905 cells were seeded in ultra-low attachment U-bottom 96-well plates. After overnight spheroid formation, CD19-, EGFR- or CD44v6 CAR T-cells or untransduced T-cells were added and cells were co-cultured for 16 h. Subsequently, (A) lysis was determined by CellTiter-Glo® 3D Viability assay and (B) Granzyme B, TNF-alpha and GM-CSF secretion of the 1:1 ratio by MACSPlex Cytotoxic T/NK cell assay. Graphs depict mean values ±SEM from at least three independent experiments.


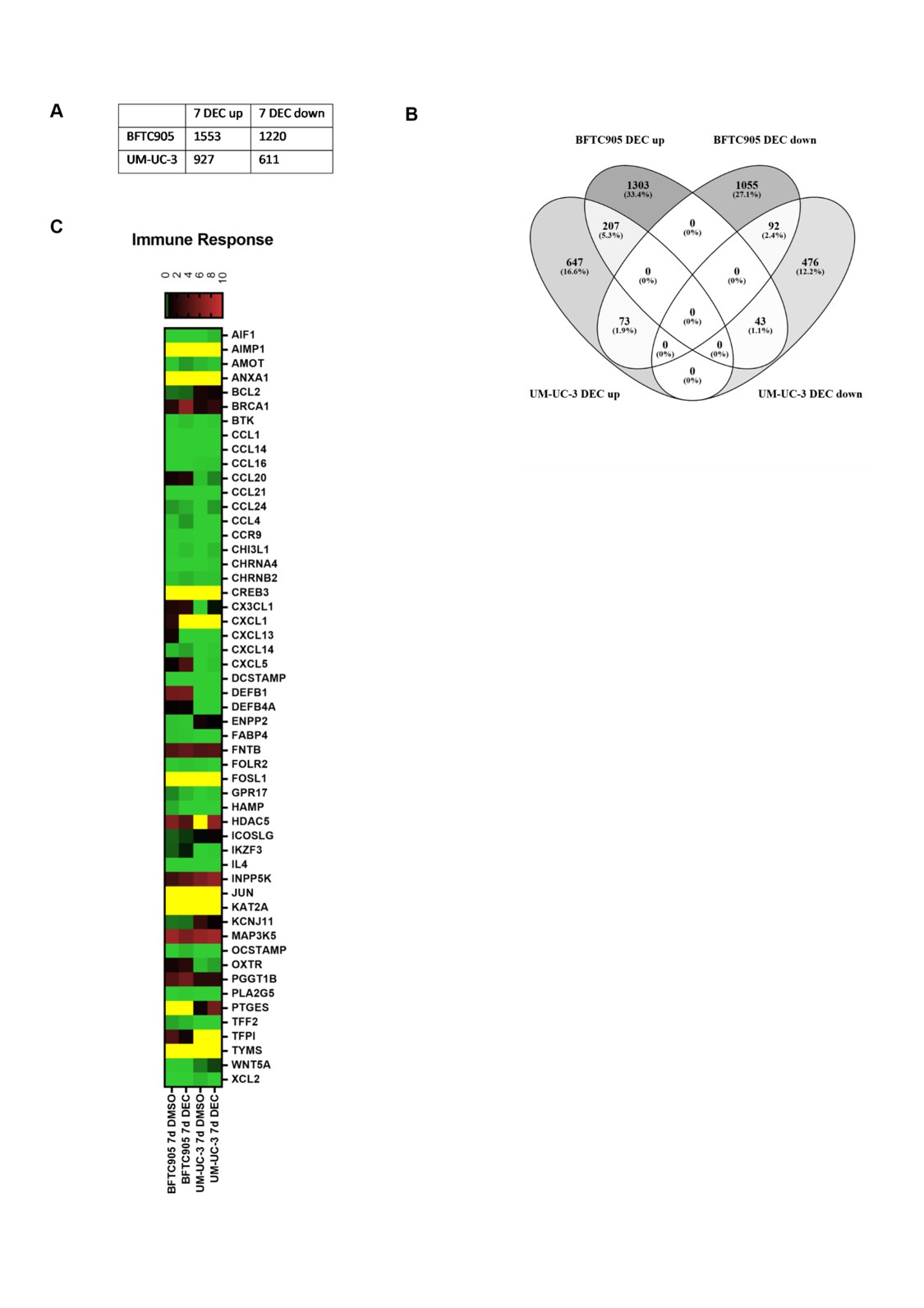
**Supplementary Figure S4.** DEC induced differential gene expression determined by RNA sequencing. (A) Number of statistical significantly differentially expressed genes seven days after treatment with DEC compared to DMSO controls. As a cut-off we applied ≥ 1.5 fold-change and p ≤0.05 after Bonferroni adjustment. (B) Commonly altered genes between the two cell lines were identified by Venn diagram analysis using the online tool Venny 2.0 ^33^. (C) RPKM expression of immune response related genes in untreated and DEC treated BFTC905 and UM-UC-3 cells displayed as heatmap. Gene lists were downloaded from the Broad Institute GSEA gene sets database [<https://www.gsea-msigdb.org/gsea/msigdb/genesets.jsp?collection=CP:KEGG>]: GO:0070098~chemokine-mediated signaling pathway, GO:0002548~monocyte chemotaxis, GO:0071356~cellular response to tumor necrosis factor, GO:0048247~lymphocyte chemotaxis, GO:0071346~cellular response to interferon-gamma, GO:0034097~response to cytokine, GO:0042113~B cell activation, GO:0006935~chemotaxis, GO:0071347~cellular response to interleukin-1. Green colored boxes indicate low expression and red colored boxes indicate high expression. Genes with expression out of range are marked in yellow.


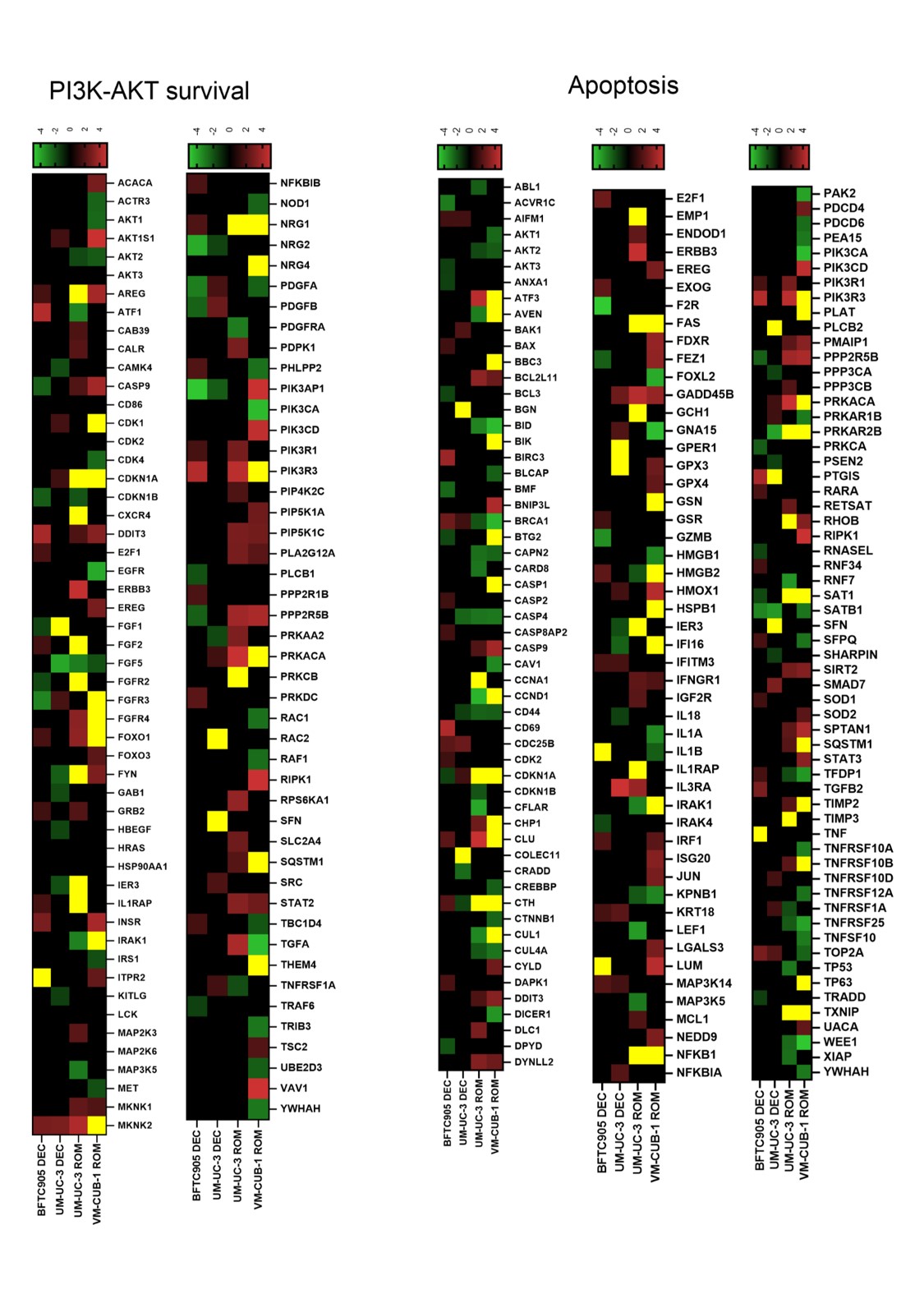
**Supplementary Figure S5.** Heatmap display of differential gene expression between untreated and epidrug treated UCC with regard to survival and apoptosis signaling. Differentially expressed genes after indicated treatment (fold-change ≥1.5) were checked for abundance in gene lists downloaded from GSEA gene set database associated with PI3K-AKT (A) and apoptosis signaling (B): HALLMARK APOPTOSIS, REACTOME EXTRINSIC PATHWAY FOR APOPTOSIS, INDUCTION OF APOPTOSIS BY EXTRACELLULAR SIGNALS, INDUCTION OF APOPTOSIS BY INTRACELLULAR SIGNALS, HALLMARK PI3K AKT MTOR SIGNALING. Fold change expression values were displayed as a heatmap with green colored boxes indicating low expression and red colored boxes indicating high expression. Genes with expression out of range are marked in yellow. Differentially expressed genes in UM-UC-3 and VM-CUB1 cells after treatment with 3 nM ROM for 72 h from our previous study (GEO accession GSE70120) were included for direct comparison.
